# Local glucocorticoid synthesis regulates house dust mite-induced airway hypersensitivity in mice

**DOI:** 10.3389/fimmu.2023.1252874

**Published:** 2023-10-23

**Authors:** Verena M. Merk, Truong San Phan, Alice Wiedmann, Rowan S. Hardy, Gareth G. Lavery, Thomas Brunner

**Affiliations:** ^1^ Department of Biology, University of Konstanz, Konstanz, Germany; ^2^ Department of Systems Immunology, Weizmann Institute of Science, Rehovot, Israel; ^3^ Institute of Clinical Sciences, University of Birmingham, Birmingham, United Kingdom; ^4^ Department of Biosciences, Nottingham Trent University, Nottingham, United Kingdom

**Keywords:** glucocorticoids, corticosteroids, 11β-hydroxysteroid dehydrogenase 1, lung, house dust mite, airway hypersensitivity, Th17, asthma

## Abstract

**Background:**

Extra-adrenal glucocorticoid (GC) synthesis at epithelial barriers, such as skin and intestine, has been shown to be important in the local regulation of inflammation. However, the role of local GC synthesis in the lung is less well studied. Based on previous studies and the uncontentious efficacy of corticosteroid therapy in asthma patients, we here investigated the role of 11β-hydroxysteroid dehydrogenase 1 (11β-HSD1/*Hsd11b1*)-dependent local GC reactivation in the regulation of allergic airway inflammation.

**Methods:**

Airway inflammation in Hsd11b1-deficient and C57BL/6 wild type mice was analyzed after injection of lipopolysaccharide (LPS) and anti-CD3 antibody, and in acute and chronic models of airway hypersensitivity induced by house dust mite (HDM) extract. The role of 11β-HSD1 in normal and inflammatory conditions was assessed by high dimensional flow cytometry, histological staining, RT-qPCR analysis, *ex vivo* tissue cultures, GC-bioassays and protein detection by ELISA and immunoblotting.

**Results:**

Here we show that lung tissue from Hsd11b1-deficient mice synthesized significantly less GC *ex vivo* compared with wild type animals in response to immune cell stimulation. We further observed a drastically aggravated phenotype in Hsd11b1-deficient mice treated with HDM extract compared to wild type animals. Besides eosinophilic infiltration, Hsd11b1-deficient mice exhibited aggravated neutrophilic infiltration caused by a strong Th17-type immune response.

**Conclusion:**

We propose an important role of 11β-HSD1 and local GC in regulating Th17-type rather than Th2-type immune responses in HDM-induced airway hypersensitivity in mice by potentially controlling Toll-like receptor 4 (TLR4) signaling and cytokine/chemokine secretion by airway epithelial cells.

## 1 Introduction

The mammalian lung epithelium and associated immune system represent an important microenvironment that controls at the same time tolerance and protection from inhaled substances or pathogens. Various mechanisms have evolved to maintain tissue homeostasis by balancing or resolving inflammation to ensure continuous and efficient oxygen exchange (1, 2). Dysregulation can lead to harmful pathologies, such as allergic asthma, which is a major burden for affected individuals and public health (3, 4). Despite serious side-effects, the efficacy of corticosteroid therapy is widely accepted and frequently applied to patients with allergic lung diseases (5, 6). The regulatory contribution of endogenous glucocorticoids (GC) to these diseases is, however, largely unknown. The adrenal glands are the main source of endogenous GC and synthesize active cortisol (humans) or corticosterone (rodents) *de novo* from cholesterol. While adrenal-derived GC are distributed throughout the body via the circulation, different extra-adrenal organs have been found to be capable of producing active GC locally, including the lungs (7, 8).While extra-adrenal GC synthesis in other epithelial barriers, especially skin and intestine, have been shown to significantly contribute to the maintenance of local immune homeostasis, tissue integrity, and thus organ functionality, the situation in the lung is poorly understood (9–15). We have previously shown that extra-adrenal lung GC synthesis is induced during acute systemic inflammation in mice, likely depending on the reactivation of inactive 11-dehydrocorticosterone (11-Dhc) to active corticosterone by 11β-hydroxysteroid dehydrogenase 1 (11β-HSD1) (16). However, the actual role of 11β-HSD1-mediated GC synthesis in immune regulation, especially during allergic airway inflammation remains unclear. We addressed this question with an *in vivo Hsd11b1* knockout model. We analyzed lung GC synthesis in wild type and knockout mice under steady state conditions, as well as upon induction of strong acute systemic inflammation. Finally, employing models of acute and chronic house dust mite (HDM)-induced airway hypersensitivity we investigated the impact of lung GC synthesis on Th1-, Th2- and Th17-types of immune responses, and associated immune cell infiltration.

## 2 Materials and methods

### 2.1 Mice

Wild type C57BL/6 and Hsd11b1-deficient (Hsd11b1^-/-^) mice were housed in specific pathogen-free (SPF) conditions in individually ventilated cages in the same room at the animal facility of the University of Konstanz. The generation of the Hsd11b1^-/-^ mice has been reported previously (17). Mice (male and female) from the same breeding stocks were used in experiments between 8-12 weeks of age. All experiments were conducted in accordance with the animal experimentation regulations of Germany and were approved by the Review Board of the Regional Council Freiburg i.B.

### 2.2 Cell culture

HEK293T cells were cultured in Dulbecco’s Modified Eagle Medium (DMEM) containing 5% steroid-free fetal bovine serum (FBS), 50 µg/ml gentamycin and 2,5 mM L-glutamine. E10 cells (murine alveolar epithelial type I cell line) were cultured in DMEM-F12 containing 10% FBS, 50 µg/ml gentamycin and 2,5 mM L-glutamine at 37°C, 5% CO_2_. E10 cells were pre-treated for 1 h with buffer control or 1 µM corticosterone, and subsequently treated with 1 µg/ml lipopolysaccharide (LPS) (S. minnesota, Sigma, St. Louis, USA) or 10 ng/ml house dust mite (HDM) (Endotoxin: 1,61 EU/µg protein, Citeq Biologics, Groningen, Netherlands) extract. Supernatant and cells were harvested at indicated time points.

### 2.3 Lipopolysaccharide and anti-CD3 antibody challenge

C57BL/6 and Hsd11b1^-/-^ mice were intraperitoneally (i.p.) injected with either 100 µl of phosphate buffered saline (PBS) (Sigma-Aldrich, St. Louis, USA), 100 µg LPS or 20 µg anti-CD3ε antibody (clone 145-2C11) in PBS. After 3 h, serum and lungs were collected and analyzed.

### 2.4 House dust mite sensitization and challenge

C57BL/6 and Hsd11b1^-/-^ mice were anesthetized with isoflurane and sensitized intranasally (i.n.) for 5 days with 100 µg (acute) or 5 days/week for 3 weeks with 25 µg (chronic) HDM extract in 50 µl PBS (25 µl/nostril). Control mice were treated with PBS. Mice were challenged with a single dose HDM extract 7 days after the last sensitization (acute) or for 5 continuous days (chronic). Mice were analyzed 24 h after the final challenge.

### 2.5 Bronchoalveolar lavage

Euthanized mice were lavaged three time with 700 µl PBS by intratracheal flexible cannula (20G, DRIFTON, Hvidovre, Denmark). Cells were spun on glass slides or stained for flow cytometry. Protease inhibitor (Roche, Basel, Switzerland) was added to the fluid (BALF) for storage.

### 2.6 Murine lung *ex vivo* culture

Lungs were cut into pieces and 200 mg were cultured in sfDMEM (5% steroid-free FBS, 50 µg/ml gentamycin, 2,5 mM L-glutamine) with or without 200 µg/ml metyrapone (MET) or 100 µM 11-dehydrocorticosterone (VWR, Radnor, USA), for 6 h (37°C, 5% CO_2_). For LPS, anti-CD3ε antibody and HDM experiments, lungs were perfused, superior lung lobes were cut and 10 mg tissue was cultured as described before. Corticosterone levels measured in MET-treated samples were subtracted to exclude contamination by serum-derived GC.

### 2.7 Luciferase-based glucocorticoid bioassay

Corticosterone levels in serum and *ex vivo* lung cultures was determined by a previously published GR-based luciferase reporter assay (16). In short, HEK293T cells were transfected with a glucocorticoid response element (GRE)-containing luciferase reporter construct (GRE2tk-LUC), a GR-expression plasmid (SVGR1) and a β-galactosidase-expression plasmid for normalization, before exposure to serum or *ex vivo* culture supernatant. Luciferase activity was determined after overnight incubation. GC concentrations were calculated using a corticosterone standard curve.

### 2.8 High dimensional flow cytometry and computational analysis

Left lung lobes were digested and single cell suspensions were obtained as previously described (18). Cells were stained with different antibody panels (**Table S2**) as follows: cells were washed twice with PBS and incubated with a fixable viability dye (**Table S2**) for 30 min at 4°C. Afterwards, cells were washed twice with staining buffer (5% BSA, 2 mM EDTA, 2 mM NaN_3_ in PBS) and incubated in blocking buffer (TruStain FcX™ Plus antibody (BioLegend, San Diego, CA, USA) in staining buffer) for 30 min at 4°C. Cells were then incubated with the respective antibodies in staining buffer for 30 min at 4°C, followed by two washing steps with staining buffer. For cytokine analysis, cells were stimulated with phorbol myristate acetate (PMA, 20 ng/ml), Ionomycin (1 µg/ml) and Brefeldin A (5 µg/ml) for 6 h prior to antibody staining. For intracellular staining, cells were fixed and permeabilized with Transcription Factor Staining Buffer Set (eBioscience, San Diego, USA) for 30 min. Samples were analyzed on a LS Fortessa (Becton Dickinson (BD), Franklin Lakes, USA). Compensation, cleaning and pre-gating for single cell, live, CD45^+^ populations was performed in FlowJo (v10.8.1). Data was exported and analyzed using an adapted R script based on Nowicka et al., 2017, Brummelman et al., 2019 and Ingelfinger et al., 2021 (19–21). FCS data were transformed (inverse hyperbolic arcsinh function) and downsampled to 2500 cells/FCS file for normalization. Combined data were applied to Uniform Manifold Approximation and Projection (UMAP) analysis (umap package v0.2.8.0) and applied to unsupervised FlowSOM clustering (FlowSOM package v2.0.0 and ConsensusClusterPlus v1.56.0). 25 to 30 clusters were, based on their marker expression, manually annotated to different myeloid und lymphoid immune cell subsets. Frequencies and total cell numbers were calculated, exported and plotted in GraphPad Prism (v.8.0; La Jolla, USA).

### 2.9 RT-qPCR

RNA isolation was performed with RNAsolv (Omega Bio-tek, Norcross, USA) or SVtotal RNA Isolation System (PROMEGA, Madison, USA) according to the manufacturer’s protocols. SYBRGreen-based RT-qPCR was performed with StepOnePlus Real-Time PCR system/QuantStudio™ 3 System (Applied Biosystems, Waltham, USA) (Primers: **Table S4**). Expression was normalized to *β-Actin.*

### 2.10 Histological analysis

Formalin-fixed and paraffin-embedded murine lung tissue sections were deparaffinized, rehydrated and hematoxylin and eosin (H&E) stained, Sirius red stained or periodic acid-Schiff (PAS) stained. For immunofluorescence, heat-induced epitope retrieval (sodium citrate pH 6,0) was performed prior to antibody staining with AlexaFluor 488-labelled anti-11β-HSD1 antibody, or isotype control (**Table S1**) (22). Cytospins were stained with Hemacolor® Rapid staining Kit (Merck, Darmstadt, Germany) according to the manufacturer’s protocol.

### 2.11 ELISA

Interleukin (IL)-4, IL-6, tumor necrosis factor (TNF) and IgE concentrations in serum, BALF or lung homogenates were quantified by ELISA according to the manufacturer’s protocols (**Table S3**).

### 2.12 Immunoblot analysis

E10 cells were harvested and lysed with radioimmunoprecipitation assay (RIPA) buffer (**Table S3**) and 40 µg protein (determined by Pierce™BCA Protein Assay Kit, Thermo Fisher, Waltham, USA) were loaded on a 12% SDS-PAGE. After electrophoretic size separation, proteins were transferred to polyvinylidene difluoride membranes (Roche). Membranes were blocked, incubated o/n at 4°C with primary antibodies (**Table S1**), and for 1 h at RT with the corresponding secondary antibody (**Table S1**) prior to development with ECL reagent (**Table S5**). Imaging was performed with ImageQuant LAS4000 (GE Healthcare, Chicago, USA).

### 2.13 Statistical analysis

Analyses were performed using GraphPad Prism (v.8.0; La Jolla, USA). Normal distribution tests: D’Agostino-Pearson omnibus, Shapiro Wilk, Kolmogorov-Smirnov test. Details are indicated in the Figure legends.

## 3 Results

### 3.1 *Hsd11b1* deletion reduces GC synthesis in the lung and causes mild changes in immune cell composition

**Figure 1:**
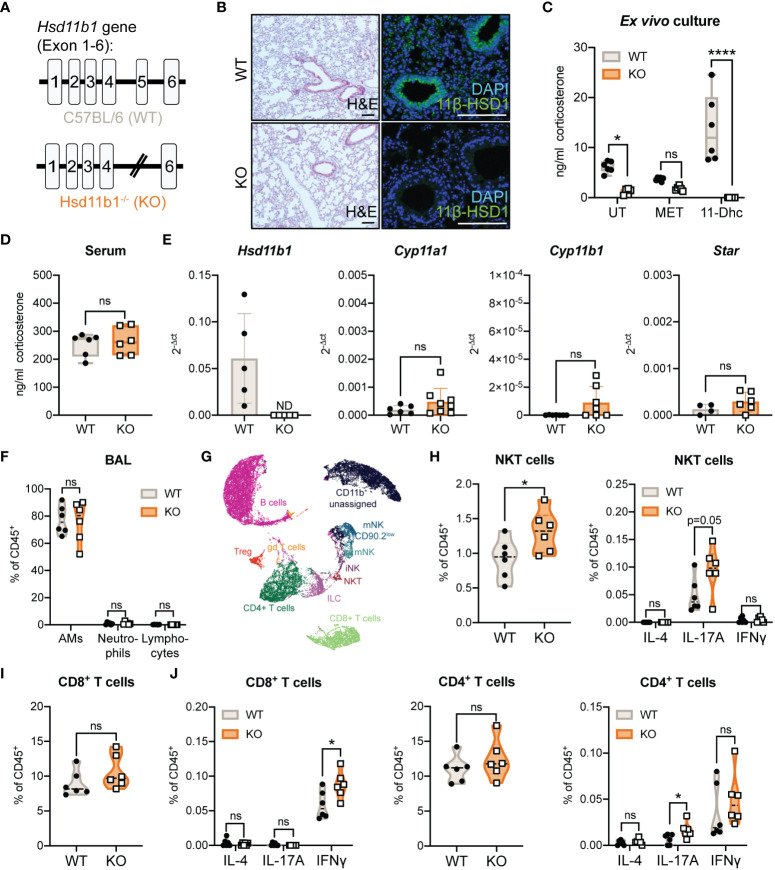
Characterization of lung GC synthesis and immune cell composition in Hsd11b1^-/-^ mice. **(A)** Schematic illustration of the *Hsd11b1* gene in C57BL/6 (wild type, WT) mice (exon 1-6), and in Hsd11b1^-/-^ (knockout, KO) mice (excision of exon 5). **(B)** Representative images of lung tissue sections stained with H&E (left panels) or anti-11β-hydroxysteroid dehydrogenase 1 (11β-HSD1) for immunofluorescence (right panels) (*n* = 12 individual animals from *n* = 2 experiments). Scale bar: 100 µm **(C, D)** Corticosterone levels in **(C)** lung *ex vivo* cultures and **(D)** serum were measured by a luciferase-based GC bioassay. *Ex vivo* cultures of WT and KO lungs were left untreated (UT), treated with 200 µg/ml metyrapone (MET) or 100 µM 11-dehydrocorticosterone (11-Dhc) for 6 (h) Dots represent individual animals (*n* = 12 individual animals from *n* = 3 experiments). **(E)** RT-qPCR analysis of *Hsd11b1*, *Cyp11a1*, *Cyp11b1* and *Star* in lungs of WT and KO mice. Expression was normalized to *β-Actin*. Bars show mean ± SD of *n* = 10-14 individual animals from *n* = 3-4 experiments. **(F-J)** Flow cytometry analysis of immune cells in **(F)** bronchoalveolar lavage (BAL) and **(G-J)** lungs of WT and KO mice. **(G)** UMAP cluster of flow cytometry data. **(H-J)** Quantification of natural killer T cells (NKT), CD4^+^ and CD8^+^ T cells (frequency of CD45^+^ cells). Dots in violin plots represent individual animals (*n* = 12 individual animals from *n* = 2 experiments). Cytokine expression (IL-4, IL-17A, IFNγ) were determined after stimulation with PMA (20 ng/ml)/Ionomycin (1 µg/ml) and BrefeldinA for 6 (h) Statistical analyses were performed using **(C, F)** two-way ANOVA, Sidak’s multiple comparisons test, **(D, E, H-J)** unpaired students T-test. ND, not detected; ns, not significant, * p<0.05, **** p<0.0001. p values are shown for p<0.1.

Our previous studies on extra-adrenal GC synthesis in murine lungs suggested a possible role of 11β-HSD1-mediated GC reactivation during acute immunological stress, but this was not investigated in a genetic model (16). Based on our observation that *Hsd11b1* is presumably expressed in all different epithelial cell types of the lung, we investigated a complete *Hsd11b1* knockout in this study (**Figure 1A**; **Supplementary Figure 1A**) (23). Firstly, we characterized constitutive lung GC synthesis in Hsd11b1^-/-^ (KO) mice in comparison with wild type (WT) animals. In KO mice, 11β-HSD1 expression was completely absent on mRNA and protein level (**Figures 1B, E**), and *ex vivo* corticosterone synthesis as well as 11-dehydrocorticosterone (11-Dhc) conversion were significantly reduced (**Figure 1C**). In contrast, serum GC levels remained unchanged (**Figure 1D**). Although 11β-HSD1 has been described to be important during lung development, we did not detect histological differences in the lung tissue (**Figure 1B**). Interestingly, we found a trend of enhanced expression of the steroidogenic enzymes *Cyp11a1*, *Cyp11b1* and the cholesterol transporter *Star*, which are important components of the *de novo* GC synthesis pathway (**Figure 1E**). The immune cell composition in lungs and bronchoalveolar lavage (BAL) of KO mice was largely unchanged (**Figures 1F-J**; **Supplementary Figures 1B-I**), however, minor differences in the frequencies of IFNγ^+^ CD8^+^, as well as IL-17A^+^ CD4^+^ T cells and natural killer T (NKT) cells indicate a certain bias towards a Th1/Th17-type differentiation in KO mice under steady-state conditions (**Figures 1H-J**).

### 3.2 Immune cell-induced GC synthesis is abolished in lungs of Hsd11b1^-/-^ mice

**Figure 2:**
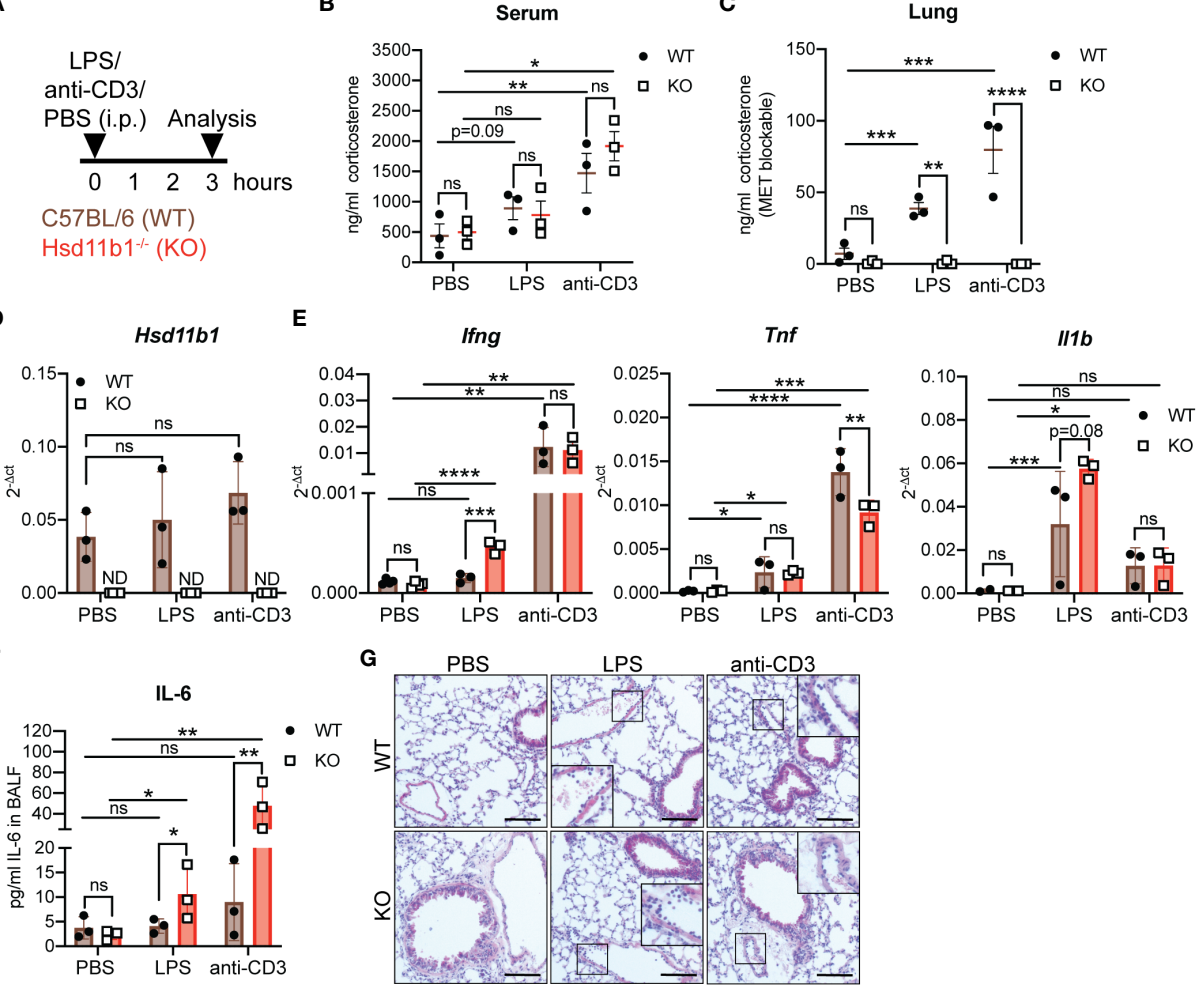
Lung *ex vivo* GC synthesis in response to acute systemic immunological stress is abolished in Hsd11b1^-/-^ mice. **(A)** Schematic overview of the experimental set up. PBS, LPS (100 µg) or anti-CD3 antibody (20 µg) were intraperitoneally (i.p) injected and wildtype (WT) and knockout (KO) mice were analyzed after 3 (h) **(B, C)** Corticosterone levels in the serum and lung *ex vivo* cultures were measured by a luciferase-based GC bioassay. Lines show mean ± SEM (*n* = 18 individual animals from *n* = 2 experiments). **(D, E)** RT-qPCR analysis for assessing *Hsd11b1*, *Ifng*, *Tnf* and *Il1b* expression in lungs. Expression was normalized to *β-Actin*. Bars show means ± SD (*n* = 18 individual animals from *n* = 2 experiments). **(F)** IL-6 concentration in bronchoalveolar lavage fluid (BALF) was determined via ELISA. Bars show means ± SD (*n* = 18 individual animals from *n* = 2 experiments). **(G)** Representative images of lung tissue sections stained with H&E (*n* = 18 individual animals from *n* = 2 experiments). Inlays show magnification. Scale bar: 100 µm. Statistical analysis was performed using **(B-E)** two-way ANOVA with Sidak’s multiple comparisons test and **(F)** multiple t tests. ND: not detected, * p<0.05, ** p<0.005, *** p<0.001, **** p<0.0001, ns, not significant. p values are shown for p<0.1.

We previously described that immune cell-induced GC synthesis in the lungs was abolished in adrenalectomized mice, suggesting that lung GC synthesis depends on adrenal precursors, and that the lung tissue converts serum-derived 11-Dhc to corticosterone in an 11β-HSD1-dependent manner (16). To test this hypothesis, we challenged WT and Hsd11b1^-/-^ (KO) mice intraperitoneally (i.p.) with lipopolysaccharide (LPS), anti-CD3 antibody or PBS as control and analyzed them after 3 h (**Figure 2A**). While we could measure an immune cell activation-induced increase in serum GC levels in both WT and KO mice (**Figure 2B**), lung GC synthesis was induced only in *ex vivo* lung cultures of WT but not of KO mice (**Figure 2C**). Both, anti-CD3 antibody and LPS injection did not result in significantly enhanced *Hsd11b1* expression but promoted, depending on the trigger, enhanced mRNA expression of inflammatory cytokines in lung tissue, i.e. *Ifng*, *Tnf* and *Il1b* (**Figures 2D, E**). Interestingly, despite short and indirect immune cell stimulation, we observed significantly more IL-6 protein in the bronchoalveolar lavage fluid (BALF) but not in liver and spleen of KO mice (**Figure 2F**; **Supplementary Figures 2A, B**). Furthermore, we observed augmented leukocyte adherence to the vasculature and extravasation, indicating initial immune cell infiltration into the lung tissue (**Figure 2G**). Overall, the results confirm the previously suggested importance of 11β-HSD1-dependent reactivation of local GC in an acute model of lung inflammation.

### 3.3 Lung inflammation is aggravated in Hsd11b1^-/-^ mice in a model of acute house dust mite airway hypersensitivity

**Figure 3:**
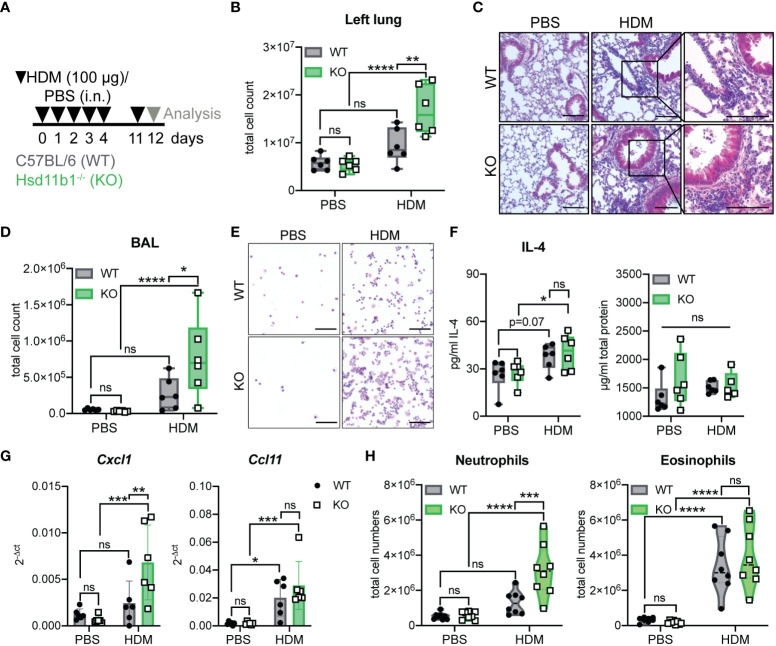
Lung inflammation is aggravated in Hsd11b1^-/-^ mice in a model of acute house dust mite (HDM) airway hypersensitivity. **(A)** Schematic overview of the experimental set up. PBS or HDM extract (100 µg) were applied intranasally (i.n.) (25 µl/nostril) to WT and KO mice for five consecutive days. Mice were challenged on day 11 and analyzed 24 h later. **(B)** Total cell counts of digested left lungs. Dots represent individual animals (*n* = 24 individual animals from *n* = 3 experiments). **(C)** Representative images of lung tissue sections stained with H&E (*n* = 24 individual animals from *n* = 3 experiments). Magnifications are shown for HDM-treated lungs. Scale bar: 100 µm. **(D)** Total cell counts in the bronchoalveolar lavage (BAL). Dots represent individual animals (*n* = 6/group). **(E)** Representative images of BAL cytospins (*n* = 24 individual animals from *n* = 3 experiments) stained with H&E. Scale bar: 100 µm. **(F)** Total protein and IL-4 in lung homogenates was determined by BCA and ELISA. Dots represent individual animals (*n* = 24 individual animals from *n* = 3 experiments). **(G)** RT-qPCR analysis for assessing *Cxcl1* and *Ccl11* expression in lung tissue. Expression was normalized to *β-Actin*. Bars show means ± SD (*n* = 24 individual animals from *n* = 3 experiments). **(H)** Flow cytometry analysis of lung neutrophils and eosinophils. Dots in violin plots represent individual animals (*n* = 32 individual animals from *n* = 4 experiments). Statistical analysis was performed by using **(B, D, F-H)** two-way ANOVA with Sidak’s multiple comparisons test. * p<0.05, ** p<0.005, *** p<0.001, **** p<0.0001, ns, not significant. p values are shown for p<0.1.

To further study the role of lung GC synthesis in the regulation of airway inflammation we continued to analyze the relevance of 11β-HSD1-mediated GC reactivation in an acute model of house-dust mite (HDM)-induced airway hypersensitivity (**Figure 3A**). Interestingly, while exposure of WT mice to HDM extract did not result in significant immune cell infiltration of lung tissue, KO mice showed significantly enhanced immune cell numbers in the lung tissue (**Figures 3B, C**; **Supplementary Figure 3A**), and the airway space, as quantified by an increased cell count in the BAL (**Figures 3D, E**; **Supplementary Figure 3B**). While total protein levels in lung homogenates of HDM treated mice remained unchanged, IL-4 was significantly enhanced in KO mice compared to control-treated animals (**Figure 3F**; **Supplementary Figure 3G**). Total serum IgE levels remained unchanged at this time point (**Supplementary Figure 3F**). Strikingly, while *Ccl11* (eosinophil chemoattractant) expression was enhanced in both WT and KO mice, in line with the observed eosinophil infiltration, elevated *Cxcl1* (neutrophil chemoattractant) expression and associated neutrophil infiltration was only observed in lungs and BAL of KO mice (**Figures 3G, H**; **Supplementary Figures 3C-E**). Interestingly, serum GC levels remained unchanged and *ex vivo* lung GC synthesis was not induced (**Supplementary Figures 3H, I**). These data indicate that the lack of basal GC reactivation in the lung of 11β-HSD1-deficient mice results in an exacerbation of acute HDM-induced allergic immune responses with a strong neutrophilic component.

### 3.4 Chronic exposure to HDM allergen further exacerbates inflammatory processes in Hsd11b1-deficient mice

**Figure 4:**
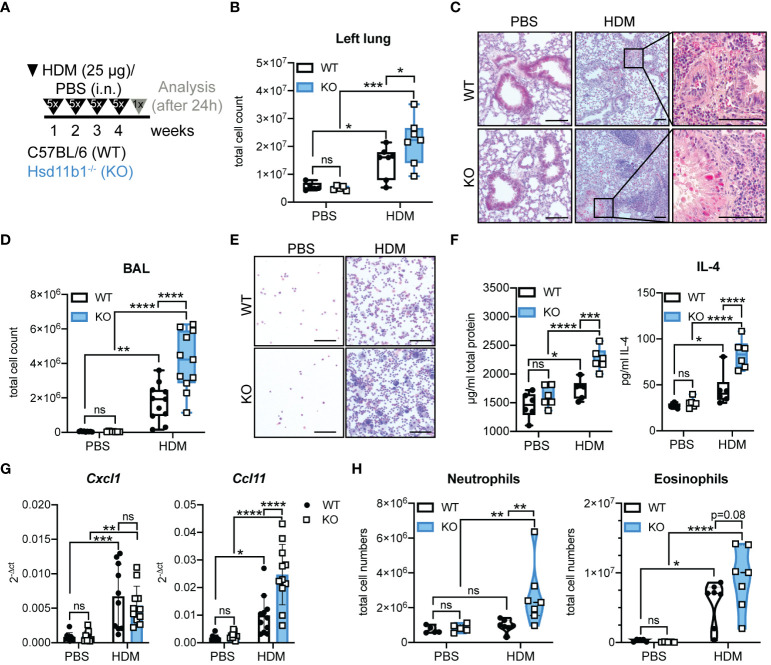
Lung inflammation is drastically aggravated in Hsd11b1^-/-^ mice in a model of chronic house dust mite (HDM) airway hypersensitivity. **(A)** Schematic overview of the experimental set up. PBS or HDM extract (25 µg) were applied intranasally (i.n.) (25 µl/nostril) to WT and KO mice for five consecutive days for four weeks. Mice were analyzed 24 h after the last challenge. **(B)** Total cell counts of digested left lungs. Dots represent individual animals (*n* = 28 individual animals from *n* = 3 experiments). **(C)** Representative images of lung tissue sections stained with H&E (*n* = 28 individual animals from *n* = 3 experiments). Magnifications are shown for HDM-treated lungs. Scale bar: 100 µm. **(D)** Total cell counts in the bronchoalveolar lavage (BAL). Dots represent individual animals (*n* = 44 individual animals from *n* = 4 experiments) **(E)** Representative images of BAL cytospins (*n* = 36 individual animals from *n* = 3 experiments) stained with H&E. Scale bar: 100 µm. **(F)** Total protein and IL-4 in lung homogenates was determined by BCA assay and ELISA. Dots represent individual animals (*n* = 24 individual animals from *n* = 3 experiments). **(G)** RT-qPCR analysis for assessing *Cxcl1* and *Ccl11* expression in lungs. Expression was normalized to *β-Actin*. Bars show means ± SD (*n* = 44 individual animals from *n* = 4 experiments). **(H)** Flow cytometry analysis of lung neutrophils and eosinophils. Dots in violin plots represent individual animals (*n* = 28 individual animals from *n* = 3 experiments). Statistical analysis was performed by using **(B, D, F-H)** two-way ANOVA with Sidak’s multiple comparisons test. * p<0.05, ** p<0.005, *** p<0.001, **** p<0.0001, ns, not significant. p values are shown for p<0.1.

In order to determine the effects of missing GC reactivation during long-term airway inflammation, mice were chronically exposed to HDM extract (**Figure 4A**). Similar to the acute model, but more pronounced, we observed a strongly aggravated phenotype of the KO animals. The immune cell infiltration into the lungs and airway space was drastically enhanced and collagen deposition and mucus production were more pronounced in HDM-treated KO mice compared to WT (**Figures 4B-E**; **Supplementary Figures 4A-E**). Additionally, total protein and IL-4 in lung homogenates and IL-4 and IL-5 mRNA expression were significantly enhanced in KO mice compared to WT mice (**Figure 4F**; **Supplementary Figure 4G**). Moreover, there was a tendency of elevated total serum IgE levels, especially in the KO mice (**Supplementary Figure 4H**). At this time point after chronic allergen exposure, *Ccl11* expression in KO lungs was significantly enhanced compared to those in WT lung, which is in line with the observed trend of enhanced eosinophil infiltration. While an allergen-induced neutrophilic infiltration was still not observed in WT lung, despite a significant increase in *Cxcl1* expression, lungs of KO mice showed a remarkable increase in neutrophil infiltration (**Figures 4G, H**; **Supplementary Figure 4F**). Similar to the acute model, serum GC levels were not increased upon HDM exposure and *ex vivo* lung GC synthesis was not induced (**Supplementary Figures 4I, J**). From these data, we concluded that the lack of basal GC reactivation results in an aggravated inflammatory phenotype and retains the characteristics of a mixed eosinophilic and neutrophilic inflammation in KO mice over time.

### 3.5 Immunophenotyping of lungs upon acute and chronic exposure to HDM allergen reveals cell type-specific differences in WT and KO mice

**Figure 5:**
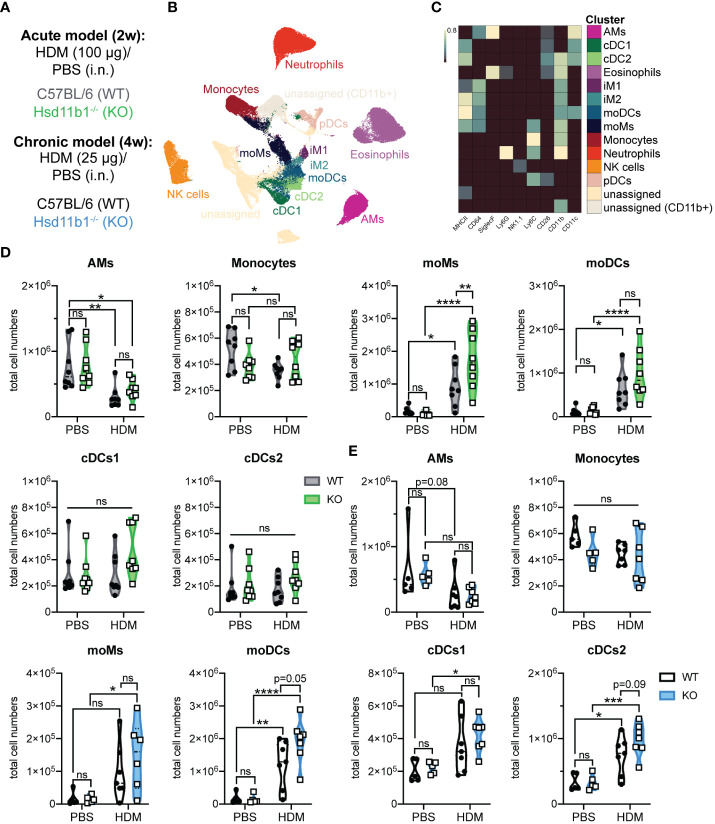
Immunophenotyping of WT and Hsd11b1^-/-^ lungs in acute and chronic house dust mite (HDM)-induced airway hypersensitivity. **(A)** Color code for the 2 weeks (2w) acute (grey/green) and 4 weeks (4w) chronic (black/blue) exposure of WT and KO mice to HDM extract. **(B)** UMAP-clustering of high dimensional flow cytometry data that was pre-gated for live/CD3^-^/CD19^-^ cells (acute model). **(C)** Heat map of FlowSOM clustering and identification of specific cell types: alveolar macrophages (AMs), conventional dendritic cells type 1,2 (cDCs1,2), eosinophils, interstitial macrophages type 1,2 (iM1,2), monocyte-derived DCs and macrophages (moDCs, moMs), monocytes, neutrophils, natural killer (NK) cells, plasmacytoid DC (pDCs). **(D, E)** Quantification of specific cell types in the **(D)** acute (*n* = 32 individual animals from *n* = 4 experiments) and **(E)** chronic (*n* = 28 individual animals from *n* = 3 experiments) model. Statistical analysis was performed by using **(D, E)** two-way ANOVA with Sidak’s multiple comparisons test. * p<0.05, ** p<0.005, *** p<0.001, **** p<0.0001, ns, not significant. p values are shown for p<0.1.

The immune response in HDM-induced airway hypersensitivity is complex and involves various cell types of the innate and adaptive immune system (24). Thus, we next expanded the immune cell analysis to additional cell types, revealing major differences in the immune cell composition between WT and KO lungs exposed to HDM extract (**Figures 5A-C**). While the number of alveolar macrophages decreased upon HDM treatment, monocytes, conventional dendritic cells type 1 (cDCs1), natural killer (NK) cells, plasmacytoid DC (pDCs) and type 1 and 2 interstitial macrophages (iMs1, 2) remained largely unchanged in both, WT and KO lungs (**Figures 5D, E**; **Supplementary Figures 5A, B**). However, the number of monocyte-derived macrophages (moMs) and monocyte-derived DCs (moDCs) drastically increased in lungs of acutely treated KO mice, indicating stronger inflammation compared to WT mice (**Figure 5D**). While this effect on moMs was less pronounced in chronically treated mice, the increase of moDCs seemed to be slightly enhanced over time, with a consistently greater increase in KO mice (**Figures 5D, E**). A similar observation was made for cDCs2, an important DC subset that contributes to Th2 and Th17 polarization of CD4^+^ T cells (**Figures 5D, E**). Taken together, these results confirm a dysregulated immune response in lungs of mice lacking GC reactivation, which is in line with the drastic phenotype of *Hsd11b1*-deficient mice in both models.

### 3.6 11β-HSD1 deficiency reinforces a Th17-type immune response

**Figure 6:**
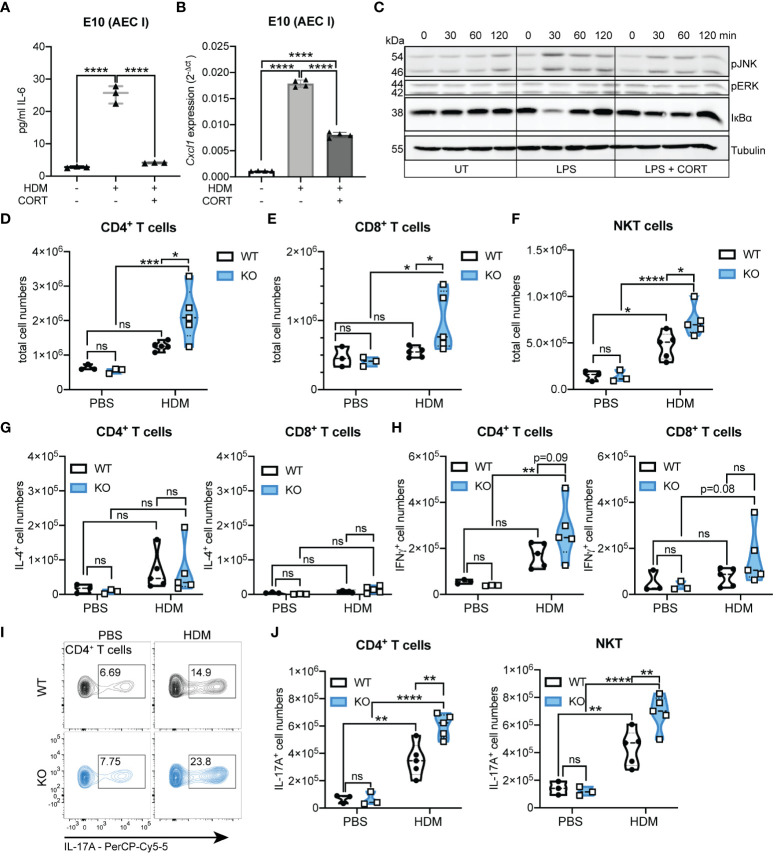
Hsd11b1 knockout reinforces the Th17-type immune response. **(A, B)** E10 (alveolar epithelial cell type I, AEC I) cells were left untreated or treated for 4 h with 35 µg/ml HDM extract with or without 1 h pre-treatment with 1 µM corticosterone. **(A)** IL-6 release into the supernatant was assessed by ELISA. Lines show means ± SD (*n* = 3 experiments). **(B)** *Cxcl1* expression was assessed by RT-qPCR. Bars show means ± SD (*n* = 4 experiments). **(C)** E10 cells were control treated or 1 h pre-treated with 1 µM corticosterone and then treated with 1 µg/ml LPS. At the indicated time points, cells were lysed and subjected to immunoblot analysis. **(D-J)** WT and KO mice were chronically exposed to HDM extract and lung cells were analyzed via flow cytometry. Quantification of **(D)** CD4^+^ T cells, **(E)** CD8^+^ T cells and **(F)** NKT cells. **(G)** IL-4 and **(H)** IFNγ producing CD4^+^ and CD8^+^ T cells. Dots in violin plots represent individual animals (*n* = 16 individual animals from *n* = 2 experiments). **(I)** Representative flow cytometry plots and **(J)** quantification of IL-17A^+^ CD4^+^ T cells and IL-17A^+^ NKT cells. Dots in violin plots represent individual animals (*n* = 16 individual animals from *n* = 2 experiments). Statistical analysis was performed by using **(A, B, D-H, J)** two-way ANOVA with Sidak’s multiple comparisons test. ** p<0.005, *** p<0.001, **** p<0.0001, ns, not significant.

Thus far, our results demonstrate that the absence of 11β-HSD1 and associated GC reactivation substantially contributes to deregulated immune cell activation and the aggravated inflammatory phenotype during HDM-induced airway hypersensitivity. The remarkable differences in neutrophil and eosinophil infiltration into the lungs of KO mice compared to WT mice indicate specific differences in their respective immune response towards HDM extract. To further characterize the underlying mechanism, we performed *in vitro* assays with an alveolar epithelial cell type 1 cell line (E10). Exposure of E10 cells to HDM extract resulted in significantly enhanced IL-6 secretion and *Cxcl1* expression (**Figures 6A, B**). This induction of inflammatory cytokines and chemokines could be suppressed when cells were pre-treated with corticosterone (**Figures 6A, B**), indicating an important role of local GC in regulating the expression of epithelial-derived cytokines and chemokines that potentially drive a Th17-type immune response. In line with this, E10 cells treated with corticosterone, prior to LPS exposure (an important component of HDM extract), showed decreased c-Jun-N-terminal kinase (JNK) and extracellular signal-regulated kinase (ERK) phosphorylation, as well as IκBα (nuclear factor of kappa light polypeptide gene enhancer in B cells inhibitor α) degradation, indicative of reduced activator protein 1 (AP-1) and NFκB (nuclear factor kappa-light-chain-enhancer of activated B cells) transcriptional activity (**Figure 6C**). In this context, we continued to analyze lymphoid immune cell subsets and their cytokine expression to determine the type of immune response in our chronic model of airway inflammation (**Figures 6D-J**; **Supplementary Figures 6A-F**). Similar to some innate immune cells, the number of CD4^+^ and CD8^+^ T cells, and NKT cells increased more drastically in KO lungs compared to WT lungs after chronic exposure to HDM extract (**Figures 6D-F**). While there was a trend for increased numbers of IL-4^+^ CD4^+^ T cells after HDM allergen exposure, no significant differences between WT and KO could be demonstrated (**Figure 6G**). In contrast, a moderate difference could be observed in IFNγ^+^ CD4^+^ T cells, but not in IFNγ^+^ or IL-17A^+^ CD8^+^ T cells. (**Figure 6H**; **Supplementary Figure 6E**). More strikingly, the analysis of IL-17A^+^ CD4^+^ T cells and IL-17A^+^ NKT cells revealed not only a massive increase of these cells in HDM-treated KO lungs, but also significant lower numbers in WT lungs (**Figures 6I, J**). In conclusion, our results demonstrate that 11β-HSD1-mediated GC reactivation regulates T cell priming and that the lack of local GC reinforces a Th17-type immune response that drives neutrophilic inflammation and associated tissue damage in HDM-induced airway hypersensitivity.

## 4 Discussion

Asthma and other allergic diseases of the lung represent a major public health burden and tremendously impair the quality of life of affected patients (3, 4). The use of synthetic corticosteroids often ameliorates acute symptoms, but does not cure or control the cause of the disease (6). In addition, various side effects upon long-term use and cases of GC resistance demand for therapeutic alternatives (6, 25). The development of novel targeted therapies often benefits from a better understanding of local immune responses in the lungs. Given the well documented role of local GC synthesis in the regulation inflammatory processes in the skin and intestine (9, 15, 26), endogenous GC synthesis in the lung may hold similar promises. It is generally accepted that GC play an important role in the regulation of lung development, and that GC treatment of preterm infants *in utero* has great therapeutic success in reducing lung disorders such as respiratory distress syndrome (RDS) (27, 28). Analysis of the expression of steroidogenic enzymes during murine fetal lung development revealed that the lung tissue is potentially capable of synthesizing bioactive GC from cholesterol, and even more interesting in the context of this study, expressing increased *Hsd11b1* mRNA levels over the gestation time (29). Furthermore, our previous study showed high *Hsd11b1* expression in adult murine lungs and provided evidence that GC synthesis in the lung is induced upon strong immunological stress in a 11β-HSD1-dependent manner (16). This was mainly concluded from the observation that the increase of *ex vivo* synthesized GC in response to anti-CD3 antibody or LPS injection was abolished in adrenalectomized mice (16). Thus 11β-HSD1-mediated reactivation may be a major mechanism of local GC synthesis in the lung. Our recent study on GC synthesis in the adult murine and human lung suggests that *de novo* GC synthesis from cholesterol exists as well, but is restricted to the large conducting airways, especially the trachea, and that 11β-HSD1-mediated GC reactivation dominates in the small conducting airways and alveoli, which correlates with the vascularization and thus substrate availability from the serum (23).

In this study we therefore aimed to assess the role of 11β-HSD1-mediated GC reactivation in the lung under homeostatic and inflammatory conditions employing a genetic model of *Hsd11b1* deletion. As expected, the general characterization of the lungs of KO mice revealed no 11β-HSD1-mediated GC reactivation. It has been previously noted that *Hsd11b1* KO mice exhibit adrenal hyperplasia, but serum corticosterone levels of non-stressed mice equal those of WT animals, which could be confirmed (17). Despite crucial functions of GC in lung development (27), we did not detect any obvious alterations in lung tissue of KO mice at the histological level. Interestingly, some enzymes of the *de novo* GC synthesis pathway tended to be increased, which might reflect a compensation reaction due to the lack of GC reactivation. However, the analysis of the steady state immune cell composition in the bronchoalveolar lavage (BAL) and within the lung tissue did not reveal major differences between WT and KO animals, even though stimulation of isolated immune cells with PMA/Ionomycin indicated, based on the cytokine pattern (30), a Th1 and Th17 bias in KO mice at basal conditions. Next to the analysis of steady-state conditions, we also addressed our previously suggested idea that 11β-HSD1 is critical for lung GC synthesis in response to immune cell activation (16). We could show that LPS- and anti-CD3-induced GC synthesis was absent in the lungs of KO mice, whereas the increase in serum GC levels was comparable between KO and WT animals. Since *Hsd11b1* mRNA expression did not increase upon immune cell stimulation, enhanced GC synthesis likely depends on the availability of serum-derived GC precursors that reach the lungs through the vascularization. LPS and anti-CD3 antibody injection are established models to induce extra-adrenal GC synthesis through increase of systemic inflammatory mediators (9, 15, 16). Although these models do not directly target the lung, we observed leukocyte adherence in blood vessels and early signs of tissue infiltration paralleled by enhanced expression of inflammatory cytokines. Even more strikingly, IL-6 levels in the BAL fluid (BALF) of KO mice were significantly increased compared to that of WT animals (**Figure 2F**). Of note, although the liver and immune cells express solid levels of *Hsd11b1* (23, 31, 32), activation-induced IL-6 expression in livers and spleens was not affected by *Hsd11b1* deletion (**Supplementary Figures 2A, B**). This indicates that 11β-HSD1, and thus local GC reactivation, is critical for balancing the immune response locally in the lung, rather than having a general impact on immune cells before they enter the lung. IL-6 is an important cytokine secreted by lung epithelial cells and immune cells, and drives Th17 polarization of T cells (33–35). The potential bias towards a Th17-type immune response in *Hsd11b1*-deficient mice becomes more persuasive when considering the analysis of acute and chronic HDM-induced airway hypersensitivity models. Although HDM-induced allergic immune reactions occurs in both WT and KO animals, the aggravated phenotype in KO mice is very obvious, and includes enhanced immune cell infiltration and tissue remodeling. The increase of IL-4 and IL-5, classical type 2 inflammation cytokines, in HDM-treated animals is expectable, however, especially IL-4 levels in lung homogenates of KO mice drastically increased over time, which further confirms an aggravated phenotype. We did not observe an effect of HDM treatment on serum corticosterone levels (**Supplementary Figures 3H**, **4I**), and no induction of *ex vivo* GC synthesis beyond the basal level (**Supplementary Figures 3I**, **4J**), suggesting a critical role of constitutive GC synthesis in the lung. This is in line with the assumption that especially barrier tissues require a tightly controlled and fine-tuned immune system to allow efficient pathogen clearance while preventing the overreaction of the immune system to non-harmful substances. Our data suggests that the lack of basal GC reactivation completely imbalances the immune response to HDM extract with drastic consequences for the pathogenic phenotype. Strikingly, while eosinophilic infiltration occurs in WT and KO lungs, neutrophilic infiltration could only be observed in KO lungs, which at least in the acute model correlated with *Cxcl1* and *Ccl11* mRNA expression. Similar results were observed for immune cells in the BAL. While under steady-state conditions, alveolar macrophages (AM) represent the majority of immune cells in the BAL, HDM treatment led to the infiltration of eosinophils in both WT and KO mice, but neutrophils only in KO mice (**Supplementary Figures 3C-E**, **4E, F**). This massive neutrophilic infiltration was, based on the relatively low endotoxin content in the HDM extract used (1,61 EU/µg protein), unexpected. It had been previously shown that Toll-like receptor 4 (TLR4) activation by LPS contaminants in HDM extracts and the subsequent release of cytokines and chemokines is indispensable for allergen sensitization, with low doses of LPS promoting a Th2-type eosinophilic immune reaction, whereas high doses of LPS rather initiate a Th17-type neutrophilic response (36–40). In line with this, direct intra-tracheal administration of LPS led to increased neutrophil infiltration into the alveolar space of KO mice (41). Interestingly, the lack of local 11β-HSD1-mediated GC reactivation does not seem to affect the type 2 phenotype in the acute HDM model, but causes a mixed type 2/type 17 phenotype with enhanced neutrophil infiltration. This potentially leads to a vicious circle of inflammation resulting in the severely exacerbated phenotype of KO mice in the chronic model, including both type 2 and type 17 characteristics. The detailed analysis of different myeloid subsets revealed that monocyte-derived antigen-presenting cells are significantly increased in KO compared with WT animals. With regard to T cell priming, conventional type 2 DC (cDCs2) are of particular interest. This CD11b^+^ CD64^-^ DC subset is more drastically elevated in KO lungs upon HDM exposure compared to WT animals. Depending on their maturation state, cDCs2 have been shown to be important to prime Th2 and Th17 differentiation in a HDM/Ovalbumin model (42, 43). The presence of IL-6 was shown to be critical for the differentiation of Th17 cells from naïve CD4^+^ T cells (33). In our experiments, the alveolar epithelial cell line E10 released IL-6 and showed enhanced expression of *Cxcl1* in response to HDM extract, which was inhibited in the presence of corticosterone. This was likely due to GC-mediated inhibition of TLR4 signaling and downstream NFκB transcriptional activity (44, 45). Detailed investigation of lymphoid cell populations and their polarization further demonstrated an enhanced Th17-type immune response in the absence of locally reactivated GC. While HDM-treated WT and KO lungs showed no differences in Th2-polarized cells, we observed a strong increase in Th17-type cells in KO lungs. Thus, the assumption that GC inhibit Th1/Th17, but not Th2 polarization, which has been established for years (44, 46), and which has been shown for other extra-adrenal organs such as skin (9), may also apply to the lung. We hypothesize that basal 11β-HSD1-mediated GC reactivation plays an important role in the shaping of the lung microenvironment under inflammatory conditions. Despite the fact that *de novo* synthesis of GC from cholesterol exists in the lung, in particular in the conducting airways (23), it can apparently not compensate for the lack of local 11β-HSD1-mediated GC reactivation during inflammatory processes of the lung, such as HDM-mediated airway hypersensitivity.

Taken together, we here demonstrated an important role of 11β-HSD1 and local GC reactivation in the regulation of inflammatory processes of the lung, particularly during HDM-induced airway hypersensitivity. We propose that in the absence of local GC synthesis unrestricted TLR4 signaling and thus enhanced cytokine and chemokine release by airway epithelial cells may result in a Th17-biased immune response, priming for neutrophilic infiltration. In addition, locally produced GC likely also impact on lung-infiltrating immune cells. Furthermore, since this mouse model of allergic airway inflammation employs a complete Hsd11b1 KO mouse, and 11β-HSD1 is also expressed by immune cells (31, 32), it cannot be ruled out that the lack of GC reactivation by immune cells further contributes to the aggravated phenotype of KO mice (47). For example, it has been shown that neutrophils upregulate *Hsd11b1* expression at early stages of acute peritonitis, potentially limiting inflammation (48). The detailed situation in lung inflammation, however, remains to be investigated. Clearly, the questions regarding the relevant cellular source of 11β-HSD1 and GC target cells needs to be further investigated in more detail employing cell type-specific Hsd11b1 KO mice. Moreover, investigating the potential role of GC on CD11b^+^ CD64^-^ DCs leading to Th17 priming will complement our understanding of the role of extra-adrenal GC synthesis in controlling inflammatory processes in the lung. Since patients with severe neutrophilic asthma are often resistant to synthetic corticosteroid therapy (24), complete elucidation of these processes could contribute to novel strategies in the therapeutic management of inflammatory lung diseases and may identify new targets for drug development.

## Data availability statement

The raw data supporting the conclusions of this article will be made available by the authors, without undue reservation.

## Ethics statement

The animal study was approved by Regierungspräsidium Freiburg i.B. The study was conducted in accordance with the local legislation and institutional requirements.

## Author contributions

VM designed the study, performed the experiments, analyzed the data, created the Figures and wrote the manuscript. TP designed the flow cytometry antibody panels and assisted with data analysis. AW assisted with the staining of some histology sections. RH and GL cooperated and provided the Hsd11b1-deficient mouse strain. TB designed the study and edited the manuscript. All authors contributed to manuscript revision, read, and approved the submitted version.
