# Supplementary material for: Local glucocorticoid synthesis regulates house dust mite-induced airway hypersensitivity in mice
Source: Front Immunol. 2023 Oct 23;14:1252874. doi: 10.3389/fimmu.2023.1252874 (PMC10626452; doi:10.3389/fimmu.2023.1252874)
Supplement: Supplementary file 1 [file DataSheet_1.pdf]

Supplementary Material

Local glucocorticoid synthesis regulates house dust mite-induced airway hypersensitivity in mice

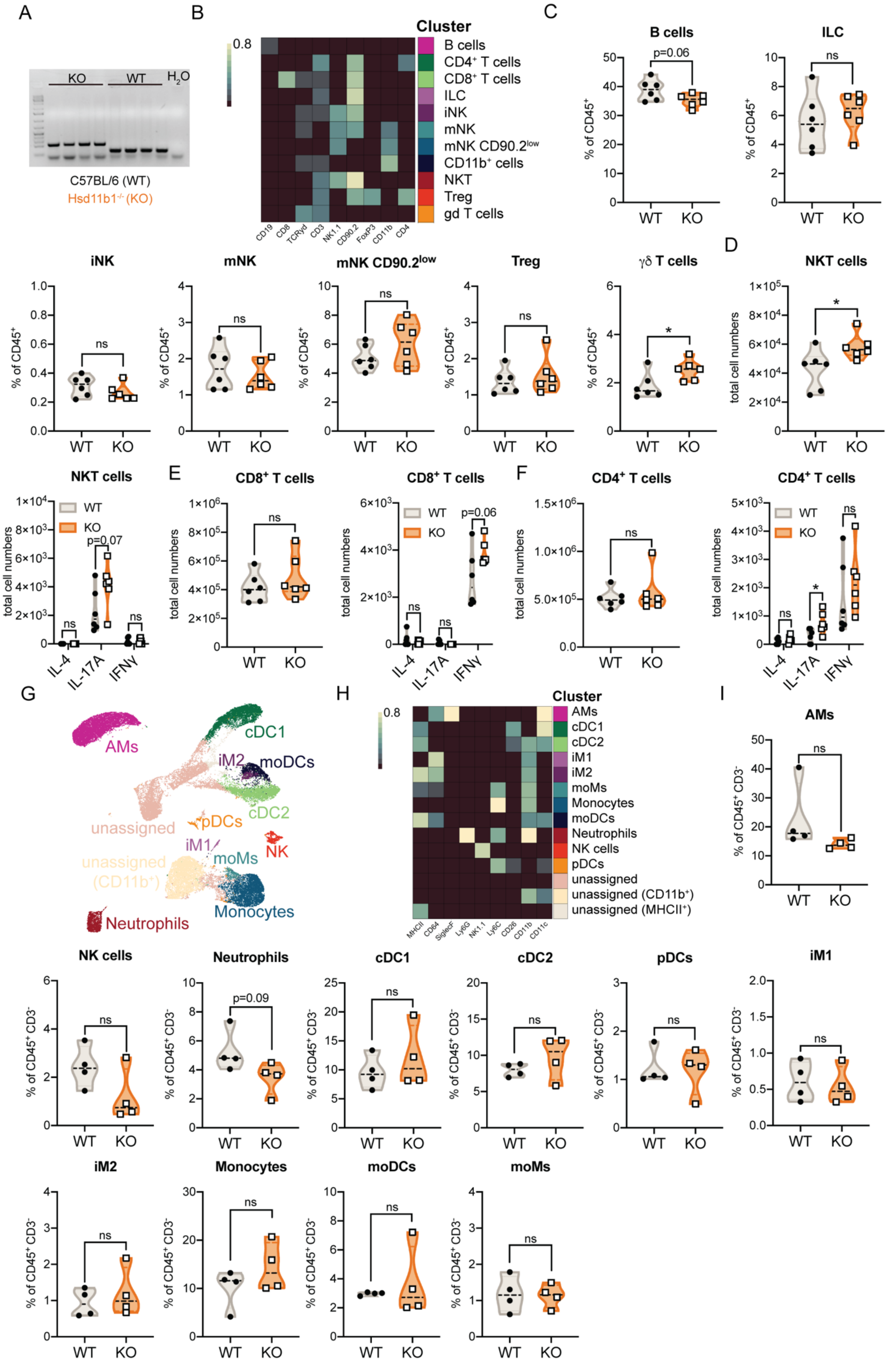
A
KO
WT
H_2O
C57BL/6 (WT)
Hsd11b1-/- (KO)
B
Cluster
0.8
B cells
CD4+ T cells
CD8+ T cells
ILC
iNK
mNK
mNK CD90.2low
CD11b+ cells
NKT
Treg
gd T cells
CD19
CD8
TCRγd
CD3
NK1.1
CD90.2
FoxP3
CD11b
CD4
C
B cells
p=0.06
ILC
ns
% of CD45+
WT
KO
iNK
mNK
mNK CD90.2low
Treg
γδ T cells
*
D
NKT cells
total cell numbers
NKT cells
IL-4
IL-17A
IFNγ
p=0.07
E
CD8+ T cells
p=0.06
F
CD4+ T cells
G
AMs
cDC1
iM2
moDCs
cDC2
unassigned
pDCs
iM1
NK
moMs
unassigned (CD11b+)
Monocytes
Neutrophils
H
Cluster
AMs
cDC1
cDC2
iM1
iM2
moMs
Monocytes
moDCs
Neutrophils
NK cells
pDCs
unassigned
unassigned (CD11b+)
unassigned (MHCII+)
MHCII
CD64
SiglecF
Ly6G
NK1.1
Ly6C
CD26
CD11b
CD11c
I
AMs
% of CD45+ CD3-
NK cells
Neutrophils
p=0.09
cDC1
cDC2
pDCs
iM1
iM2
Monocytes
moDCs
moMs

Supplementary Figure 1. Characterization of immune cell subsets in untreated WT and Hsd11b1$^{-/-}$ mice. (A) Agarose gel displaying the confirmation of *Hsd11b1* deletion via PCR. WT: wild type, KO: knockout. (B-I) Computational analysis of high-dimensional flow cytometry data from untreated WT and KO mice. (B) Heat map of FlowSOM clustering and identification of specific cell types: B cells, CD4$^+$ T cells, CD8$^+$ T cells, innate lymphoid cells (ILC), immature (i) and mature (m) natural killer (NK) cells, natural killer T (NKT) cells, regulatory T cells (Treg), γδ T cells (gd T cells). (C) Quantification, dots represent individual animals, violin plots show frequency of live, CD45$^+$ of $n = 12$ individual animals from $n = 2$ experiments. (D-F) Total cell numbers of NKT cells, CD8$^+$ T cells and CD4$^+$ T cells including IL-4$^+$, IL-17A$^+$ and IFNγ$^+$ cells. Data corresponds to main Figure 1 H-J. (G, H) UMAP clusters and heat map of FlowSOM clustering and identification of specific cell types: alveolar macrophages (AMs), NK cells, neutrophils, conventional dendritic cells type 1, 2 (cDCs1,2), interstitial macrophages type 1, 2 (iM1,2), monocytes, monocyte-derived DCs (moDCs) and macrophages (moMs), plasmacytoid DC (pDCs). (I) Quantification, dots represent individual animals, violin plots show frequency of live, CD45$^+$, CD3$^-$ of $n = 8$ individual animals from $n = 2$ experiments. Statistical analysis was performed by using (C-F, I) unpaired student's t-test. P value is displayed if $p<0.1$, * $p< 0.05$, ns: not significant.

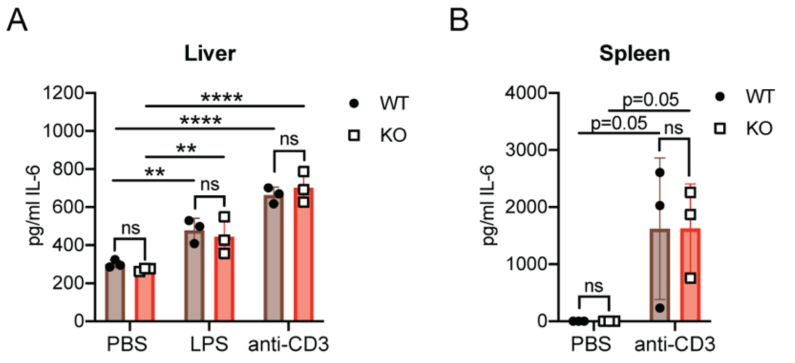

Supplementary Figure 2. Hsd11b1-deficiency does not alter IL-6 production in liver and spleen upon strong immunological stress. (A, B) PBS, LPS (100 µg) or anti-CD3 antibody (20 µg) were intraperitoneally (i.p) injected and wildtype (WT) and knockout (KO) mice were analyzed after 3 h. IL-6 concentrations in (A) liver ($n = 18$ individual animals from $n = 2$ experiments) and (B) spleen ($n = 12$ individual animals from $n = 2$ experiments) were determined via ELISA. Bars show means ± SD. Statistical analysis was performed using two-way ANOVA with Sidak's multiple comparisons test. ** $p<0.005$, **** $p<0.0001$. ns: not significant. P values are shown for $p<0.1$

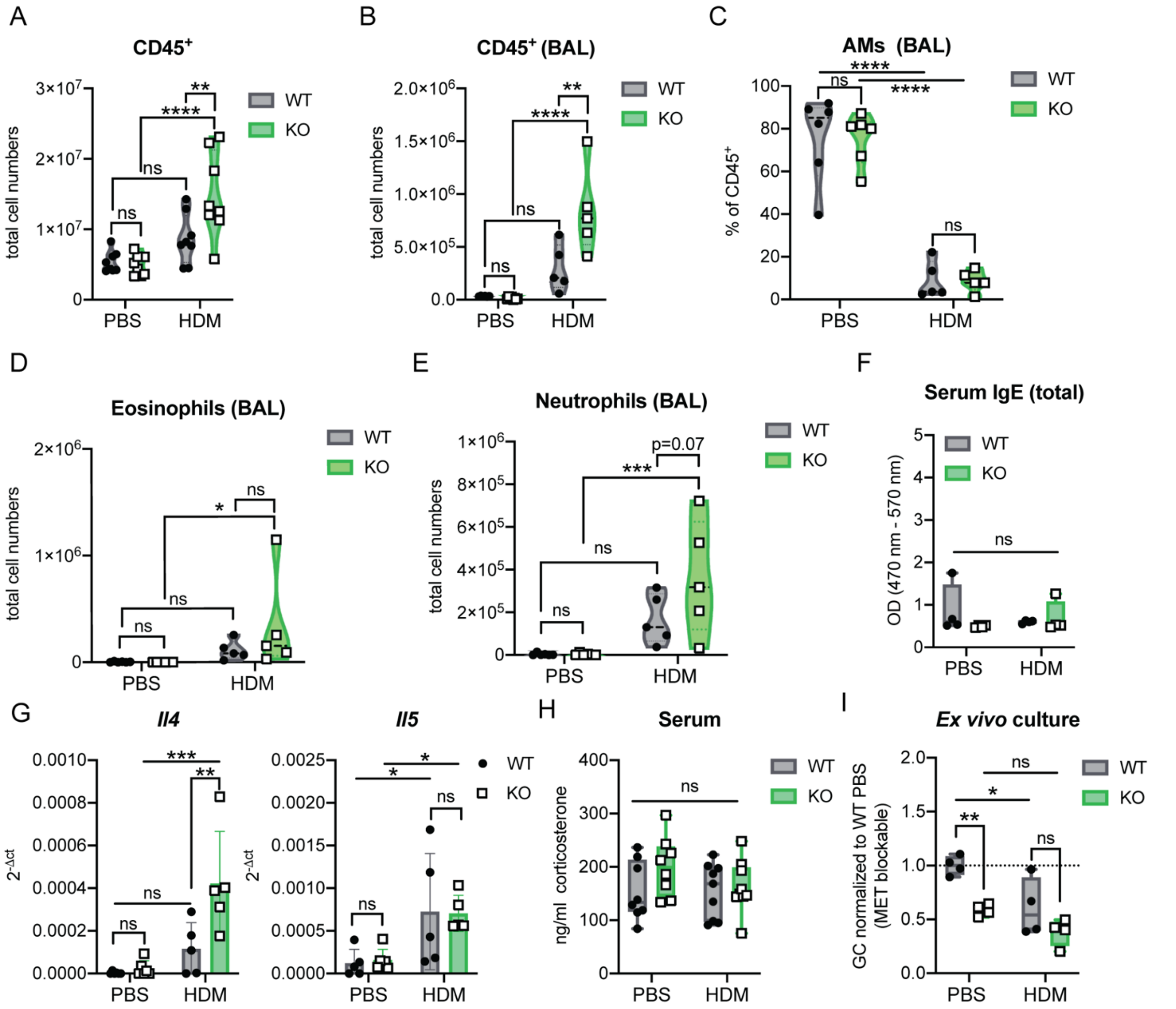

Supplementary Figure 3. Acute model of HDM-induced airway hypersensitivity. (A-E) Quantification of flow cytometry analysis of (A) lung tissue (*n* = 32 individual animals from *n* = 4 experiments) and (B-E) bronchoalveolar lavage (BAL) cells (*n* = 22 individual animals from *n* = 3 experiments) from WT and KO mice acutely treated with HDM extract. Violin plots display (A) total cell number of CD45$^+$ cells in lung tissue, (B) total cell number of CD45$^+$ cells in BAL, (C) frequency of live, CD45$^+$ alveolar macrophages (AMs), (D) total cell numbers of eosinophils, (E) total cell numbers of neutrophils. (F) Analysis of total IgE in serum by ELISA. Dots represent individual animals (*n* = 16 individual animals from *n* = 2 experiments). OD: optical density. (G) Expression of *Il4* and *Il5* in lung tissue determined by RT-qPCR. Dots represent individual animals and bars show mean $\pm$ SD of (*n* = 20 individual animals from *n* = 3 experiments). (F, G) Corticosterone level in (H) serum (*n* = 32 individual animals from *n* = 4 experiments) and (I) lung *ex vivo* cultures (*n* = 16 individual animals from *n* = 2 experiments) were determined by a luciferase-based GC bioassay. Dots represent individual animals. (A-I) Statistical analysis was performed by using two-way ANOVA with Sidak's multiple comparisons test. * $p< 0.05$, ** $p< 0.005$, *** $p<0.001$, **** $p<0.0001$, ns: not significant.

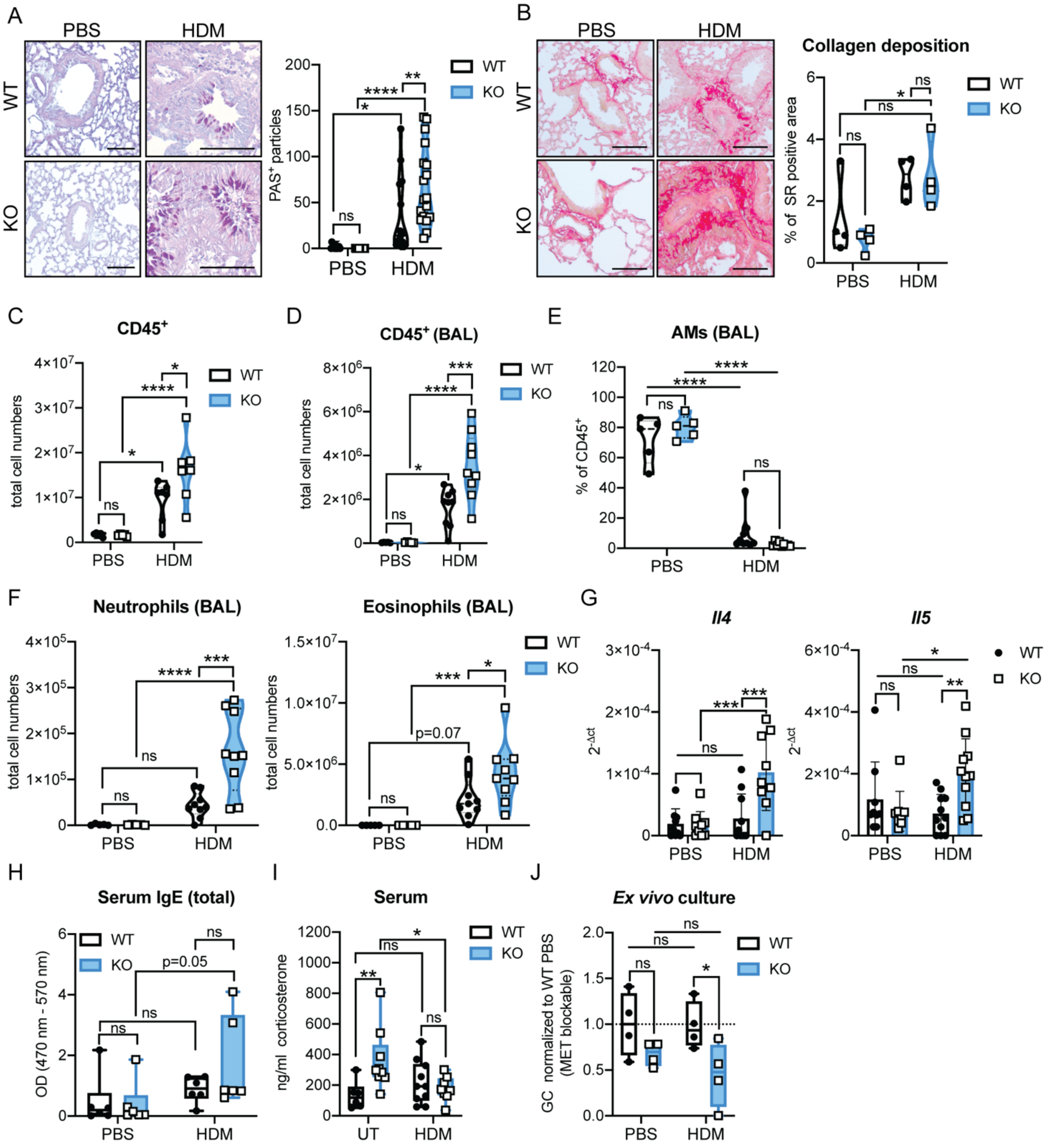

Supplementary Figure 4. Chronic model of HDM-induced airway hypersensitivity. (A) Mucus production in WT and KO mice chronically treated with HDM extract was determined by PAS staining (Table S5). Quantification was performed in ImageJ, violin plots show PAS$^+$ particles of seven bronchioles ($n = 12$ individual animals from $n = 3$ experiments). Scale bar: 100 µm. (B) Collagen deposition was determined by Sirius Red (SR) staining (Table S5) and Quantification was performed in ImageJ. Violin plots show % of SR positive area ($n = 16$ individual animals from $n = 3$ experiments). Dots represent mean of 5 bronchioles/mouse. (C-F) Quantification of flow cytometry analysis of (C) lung tissue ($n = 28$ individual animals from $n = 3$ experiments) and (D-F) bronchoalveolar lavage

(BAL) cells ($n = 36$ individual animals from $n = 3$ experiments). Violin plots display (C) total cell number of CD45^{+} cells in lung tissue, (D) total cell number of CD45^{+} cells in BAL, (E) frequency of live, CD45^{+} alveolar macrophages (AMs), (F) total cell numbers of neutrophils and eosinophils. (G) Expression of *Il4* and *Il5* in lung tissue determined by RT-qPCR. Dots represent individual animals and bars show mean $\pm$ SD ($n = 36$-44 individual animals from $n = 3$-4 experiments). (H) Analysis of total serum IgE levels by ELISA. Dots represent individual animals ($n = 24$ individual animals from $n = 3$ experiments). (I, J) Corticosterone level in (I) serum ($n = 32$ individual animals from $n = 4$ experiments) and (J) lung *ex vivo* cultures ($n = 16$ individual animals from $n = 2$ experiments) were determined by a luciferase-based GC bioassay. Dots represent individual animals. (A-J) Statistical analysis was performed by using two-way ANOVA with Sidak's multiple comparisons test. * p< 0.05, ** p<0.005, *** p<0.001, **** p<0.0001, p value is displayed if p< 0.1, ns: not significant.

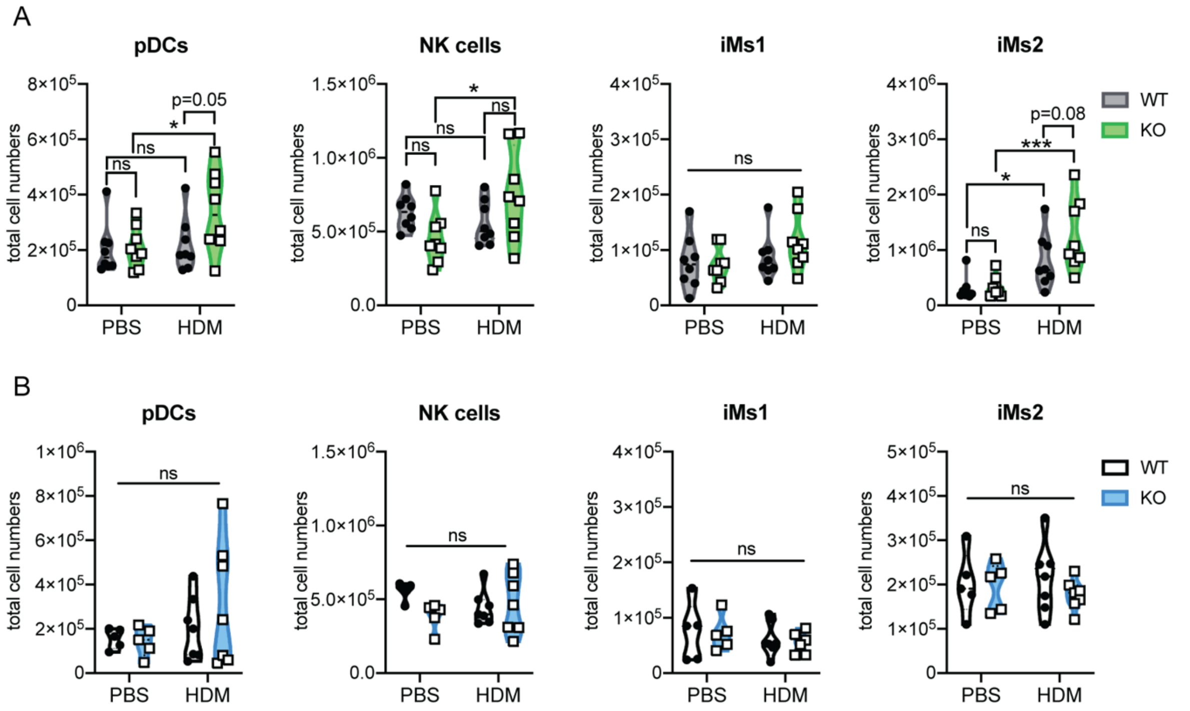

Supplementary Figure 5. Immunophenotyping in lungs of acutely and chronically HDM-treated WT and KO mice. (A, B) Quantification of high dimensional flow cytometry analysis from (A) acutely ($n = 32$ individual animals from $n = 4$ experiments) and (B) chronically ($n = 28$ individual animals from $n = 3$ experiments) HDM-treated WT and KO mice. Dots represent individual animals, violin plots display total cell numbers of plasmacytoid DCs (pDCs), natural killer (NK) cells and interstitial macrophages type 1,2 (iM1,2). Statistical analysis was performed by using two-way ANOVA with Sidak's multiple comparisons test. * p< 0.05, *** p<0.001, p value is displayed if p< 0.1, ns: not significant.

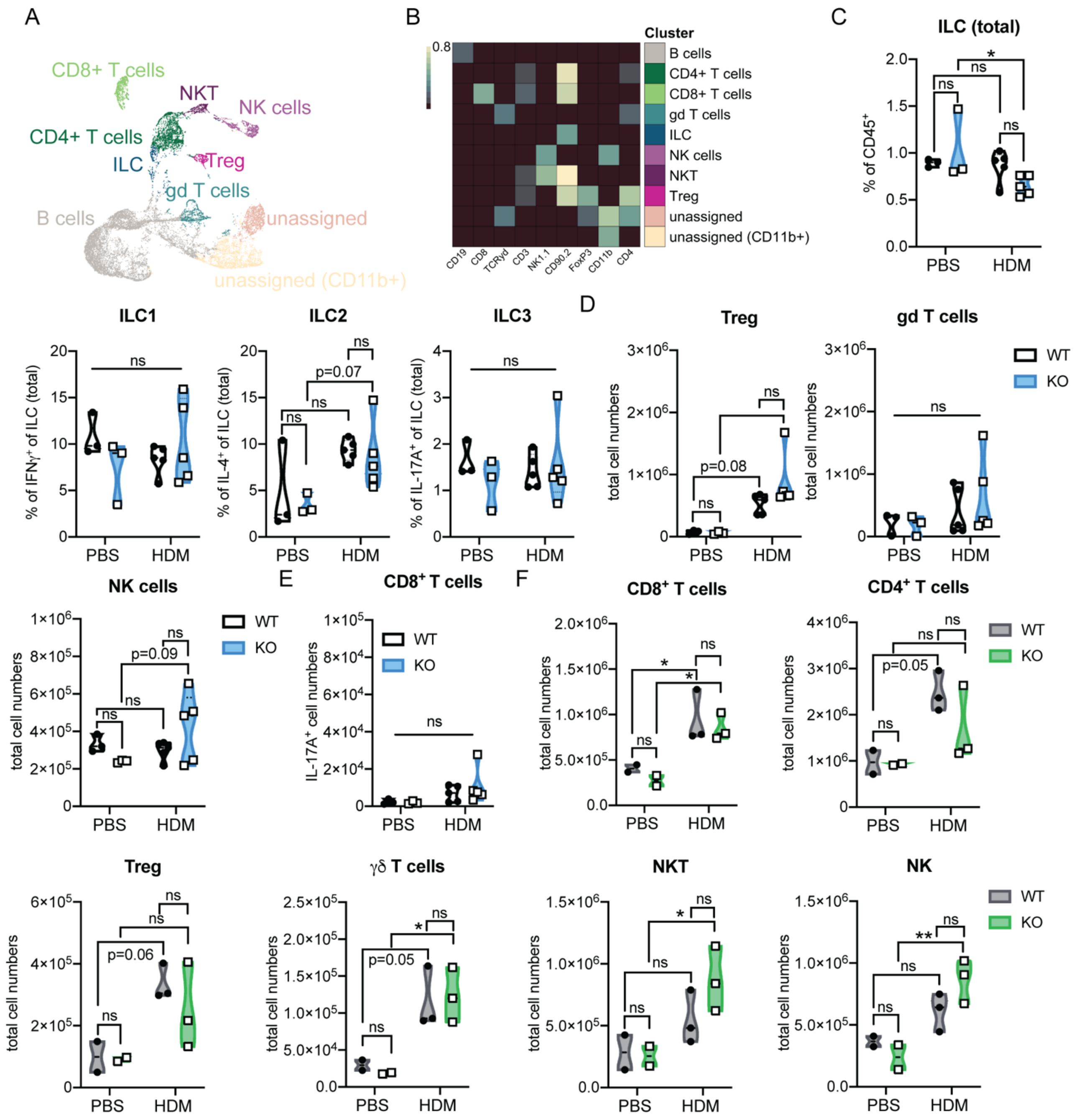

Supplementary Figure 6. Immunophenotyping (lymphoid subsets) in lungs of acutely and chronically HDM-treated WT and KO mice. (A, B, D-F) Computational analysis of high-dimensional flow cytometry data from (C-E) chronically ($n = 16$ individual animals from $n = 2$ experiments) and (F) acutely ($n = 10$ individual animals from $n = 1$ experiment) HDM-treated WT and KO mice. (A) UMAP cluster and (B) heat map of FlowSOM clustering to identify specific cell types: B cells, CD4$^+$ T cells, CD8$^+$ T cells, innate lymphoid cells (ILC), natural killer (NK) cells, natural killer T (NKT) cells, regulatory T cells (Treg), γδ T cells (gd T cells). (C) Manually gated innate lymphoid cell (ILC) populations. Total ILC are displayed as % of live, CD45$^+$ cells, ILC1 as % of IFNγ$^+$ ILCs, ILC2 as % of IL-4$^+$ ILCs and ILC3 as % of IL-17A$^+$ ILCs. (D-F) Violin plots display total cell numbers. (E) Total cell numbers of IL-17A+ CD8+ T cells. (C-F) Dots represent individual animals. Statistical

analysis was performed by using two-way ANOVA with Sidak's multiple comparisons test. * $p < 0.05$, p value is displayed if $p < 0.1$, ns: not significant.

Supplementary Table 1. Antibody list (unlabeled)

Antibody	CAT	Supplier
anti-CD3ε (clone 145-2C11)	100302	BioLegend
Rabbit anti-phospho JNK	4671	Cell-Signaling
Rabbit anti-ikB	9242	Cell-Signaling
Rabbit anti-phopho ERK	4376	Cell-Signaling
Mouse anti-Tubulin	T5168	Sigma Aldrich
Rabbit anti-11β-HSD1	ab39364	Abcam
Biotin-SP-AffiniPure Goat anti-rabbit IgG	111-065-144	Jackson ImmunoResearch Laboratories, Inc.
Peroxidase-AffiniPure Goat anti-rabbit IgG	111-035-003	Jackson ImmunoResearch Laboratories, Inc.
Peroxidase-AffiniPure Goat anti-mouse-IgG	115-035-174	Jackson ImmunoResearch Laboratories, Inc.

Supplementary Table 2. Flow cytometry antibodies (labeled)

Antibody	CAT	Supplier
Panel #1		
FVD455eFluor™UV	65-0868-14	eBioscience
CD45-FTIC	11-0451-85	eBioscience
CD11b-PECy7	101216	BioLegend
CD3-BV605	100237	BioLegend

CD19-APC-Fire750	115557	BioLegend
CD4-PE-Dazzle594	100455	BioLegend
CD8-BV421	100737	BioLegend
NK1.1-BV711	108745	BioLegend
CD90.2-BV786	105331	BioLegend
TCRgd-BV510	118131	BioLegend
FOXP3-PE	12-5773-80	eBioscience
IFNγ-APC or IL-4-APC	505809/ 504105	BioLegend
IL-17A-PerCP-Cy5.5	45-7177	BioLegend
Panel #2		
FVD455eFluor™UV	65-0868-14	eBioscience
CD26-FITC	137805	BioLegend
Ly-6G-BV605	127639	BioLegend
Ly-6C-BV785	128041	BioLegend
NK1.1-BV711	108745	BioLegend
CD11b-PECy7	101216	BioLegend
Siglec-F-BV421	565934	BD Horizon™
CD64-AlexaFluor647	139321	BioLegend
CD45-AlexaFluor700	103128	BioLegend

CD11c-PE eFluor610	61-0114-82	eBioscience
MHCII-APC eFluor780	47-5321-82	eBioscience
CD19-eFluor506	69-0193-80	eBioscience
CD3-eFluor506	69-0032-80	eBioscience

Supplementary Table 3. ELISA antibodies

**Antibody**	**CAT**	**Supplier**
IL-6	#88-7064-88	Invitrogen
IL-4	#504201 #504101	BioLegend
IgE	#406903 #406902	BioLegend

Supplementary Table 4. PCR primers

**Target**	**Primer sequence**	**Application**
Il4	Fwd:TCACAGCAACGAAGAACACCA<br>Rev:CAGGCATCGAAAAGCCCGAA	RT-qPCR
Il5	Fwd:AGGCTTCCTGTCCCTACTCAT<br>Rev:CCTCGCCACACTTCTCTTTTTG	RT-qPCR
Hsd11b1	Fwd:GCTCCCTACTCTGCAAGCAA<br>Rev:ACACCTCGCTTTTGCGTAGA	RT-qPCR
Cyp11b1	Fwd:GCCATCCAGGCTAACTCAAT<br>Rev:CATTACCAAGGGGGTTGATG	RT-qPCR

Cyp11a1	Fwd:TGCCTGGGATGTGATTTTC<br>Rev:GGGTACTGGCTGAAGTCTCG	RT-qPCR
bactin	Fwd:GATCAAGATCATTGCTCCTCCTGA<br>Rev:CAGCTCAGTAACAGTCCGCC	RT-qPCR
Il6	Fwd:CACAAGTCCGGAGAGGAGAC<br>Rev:TTGCCATTGCACAACTCTTT	RT-qPCR
Star	Fwd:TGGATGGGTCAAGTTCGACG<br>Rev:TCCTCTGCAGGACCTTGATCT	RT-qPCR
Tnf	Fwd:CCACATCTCCCTCCAGAAAA<br>Rev:AGGGTCTGGGCCATAGAACT	RT-qPCR
Il1b	Fwd:TGAGTCGGCAAAGAAATCAAGATG<br>Rev:AGAGAGAGATGGTCAATGGCAG	RT-qPCR
Ifng	Fwd:TATTGCCAAGTTTGAGGTCAACAA<br>Rev:TGGTGGACCACTCGGATGA	RT-qPCR
Cxcl1	Fwd:TCCAGAGCTTGAAGGTGTTGCC<br>Rev:AACCAAGGGAGCTTCAGGGTCA	RT-qPCR
Ccl11	Fwd:TCCATCCCAACTTCCTGCTGCT<br>Rev:CTCTTTGCCCAACCTGGTCTTG	RT-qPCR
Hsd11b1	Fwd:CTGGGAGCTTGCTTACAGCATCA<br>Rev:CATTCTCAAGGTAGATTGAACTCTG<br>Rev:AGTCCATGCAATCAACTTCTCGTC	Genotyping

Supplementary Table 5. Buffer compositions

Buffer	Ingredients
RIPA (radioimmunoprecipitation assay buffer)	50 mM Tris, 150 mM NaCl, 0.1% SDS, 0.5% sodium deoxycholate, 1% NP40 in H2O, pH 7.8
ECL	2.5 mM Luminol, 0.4 mM p-Coumaric acid, 10 mM Tris, 0.015% H2O2 in H2O, pH 8.5
Sirius Red Staining	#365548 Direct Red 80 Sigma-Aldrich<br># P6744 Picric acid (1,3% in H_2O) Sigma-Aldrich
PAS Staining	#375810 0,5% periodic acid solution<br>#3952016 Schiffs reagent
